# Validity and Reliability of Hydraulic-Analogy Bioenergetic Models in Sprint Roller Skiing

**DOI:** 10.3389/fphys.2021.726414

**Published:** 2021-09-13

**Authors:** Julius Lidar, Erik P. Andersson, David Sundström

**Affiliations:** ^1^Department of Quality Management and Mechanical Engineering, Sports Tech Research Centre, Mid Sweden University, Östersund, Sweden; ^2^Department of Health Sciences, Swedish Winter Sports Research Centre, Mid Sweden University, Östersund, Sweden; ^3^Faculty of Health Sciences, School of Sport Sciences, UiT The Arctic University of Norway, Tromsø, Norway

**Keywords:** bioenergetics, cross-country skiing, metabolism, anaerobic, aerobic, critical power

## Abstract

**Purpose:** To develop a method for individual parameter estimation of four hydraulic-analogy bioenergetic models and to assess the validity and reliability of these models’ prediction of aerobic and anaerobic metabolic utilization during sprint roller-skiing.

**Methods:** Eleven elite cross-country skiers performed two treadmill roller-skiing time trials on a course consisting of three flat sections interspersed by two uphill sections. Aerobic and anaerobic metabolic rate contributions, external power output, and gross efficiency were determined. Two versions each (fixed or free maximal aerobic metabolic rate) of a two-tank hydraulic-analogy bioenergetic model (2TM-fixed and 2TM-free) and a more complex three-tank model (3TM-fixed and 3TM-free) were programmed into MATLAB. The aerobic metabolic rate (*MR*_*ae*_) and the accumulated anaerobic energy expenditure (*E*_*an,acc*_) from the first time trial (STT1) together with a gray-box model in MATLAB, were used to estimate the bioenergetic model parameters. Validity was assessed by simulation of each bioenergetic model using the estimated parameters from STT1 and the total metabolic rate (*MR*_*tot*_) in the second time trial (STT2).

**Results:** The validity and reliability of the parameter estimation method based on STT1 revealed valid and reliable overall results for all the four models vs. measurement data with the 2TM-free model being the most valid. Mean differences in model-vs.-measured *MR*_*ae*_ ranged between -0.005 and 0.016 kW with typical errors between 0.002 and 0.009 kW. Mean differences in *E*_*an,acc*_ at STT termination ranged between −4.3 and 0.5 kJ and typical errors were between 0.6 and 2.1 kJ. The root mean square error (RMSE) for 2TM-free on the instantaneous STT1 data was 0.05 kW for *MR*_*ae*_ and 0.61 kJ for *E*_*an,acc*_, which was lower than the other three models (all *P* < 0.05). Compared to the results in STT1, the validity and reliability of each individually adapted bioenergetic model was worse during STT2 with models underpredicting *MR*_*ae*_ and overpredicting *E*_*an,acc*_ vs. measurement data (all *P* < 0.05). Moreover, the 2TM-free had the lowest RMSEs during STT2.

**Conclusion:** The 2TM-free provided the highest validity and reliability in *MR*_*ae*_ and *E*_*an,acc*_ for both the parameter estimation in STT1 and the model validity and reliability evaluation in the succeeding STT2.

## Introduction

In endurance sports, performance is highly dependent on available energy and metabolic rate. Muscle contraction is powered by the release of energy through the breakdown of adenosine triphosphate (ATP), the stores of which are replenished in three major ways, (1) through the alactic phosphagen system, (2) through the lactic glycolytic system, and (3) through the mitochondrial respiration (see e.g., Baker et al., 2010 for a thorough review). Mitochondrial respiration, also known as aerobic metabolism, can be measured through indirect calorimetry. However, the remaining two energy systems (also referred to as anaerobic systems) are not easily measured, although they often play a key role in many sports. During variable intensity exercises, such as football, road cycling, and cross-country skiing, the anaerobic energy stores might be significantly depleted from high-intensity bouts and replenished during low-intensity exercise (Buchheit et al., 2012; Skiba et al., 2012; Gløersen et al., 2020). This discharge-recharge of the anaerobic stores occurs with varying time intervals and exercise intensities throughout entire sports activities. Since only the aerobic metabolic rate is easily measured, a bioenergetic model is needed to relate the expenditure level of the anaerobic stores to the aerobic metabolic rate and power output.

Several models that include both anaerobic expenditure and recovery have been proposed throughout the years, most of which relate to the critical power concept (Sreedhara et al., 2019). The concept of critical power was introduced as a local muscle work capacity model (Monod and Scherrer, 1965), but was later extended to full body work (Moritani et al., 1981). The critical power concept simplifies the human bioenergetic system into two components: a finite aerobic metabolic rate (referred to as the critical power) and a finite anaerobic work capacity. With the critical power concept, the power demand of the working muscles is supplied by the aerobic metabolic pathway up to the critical power threshold. At power outputs above critical power, the anaerobic stores will be utilized to match the power demand until they are depleted, at which time the power output becomes limited to the critical power. According to the critical power concept, the power output corresponding to the critical power can be maintained indefinitely. However, due to premature fatigue, critical power cannot be practically maintained longer than ∼20–80 min (Hill and Smith, 1999).

Despite some of its apparent flaws, e.g., that aerobic power at the critical power threshold is instantly available at the onset of exercise (Morton, 2006), the critical power concept has been widely used in research, with some attempts on using intermittent protocols (Sreedhara et al., 2019). Moreover, the critical power concept was adapted by Morton and Billat (2004) for intermittent exercise above and below critical power, respectively. In their model, the anaerobic recovery rate was given by the difference between critical power and the current power output, which implies a constant anaerobic recovery rate at a constant power output. Conversely, Ferguson et al. (2010) presented evidence that the anaerobic work capacity recovers in a curvilinear fashion and also is not solely dependent on either oxygen uptake ($\dot{V}\,O_{2}$) or lactate levels. In line with these findings, Skiba et al. (2012) refined the intermittent critical power model to include exponential recovery of the anaerobic work capacity as a function of time, but also dependent on the power output below critical power and the level of expended anaerobic resources. The dependency on power output below critical power was empirically derived. Later, the model was reformulated with the empirically derived dependency being replaced by an algebraically derived dependency also dependent on the total anaerobic work capacity (Skiba et al., 2015). However, in the model of Skiba et al. (2015) the aerobic metabolic rate is equal to the critical power from the onset of exercise, hence no insight on the variation of aerobic metabolic rate is considered. Bartram et al. (2018) examined the validity of the model for elite cyclists and found that it underestimated the anaerobic recovery rate. Although the model could be applied to arbitrary varying power output, this has, to our knowledge, not been tested.

Morton (2006) developed a hydraulic conceptualization model where both aerobic and anaerobic energy systems are represented by fluid volumes in separate tanks and the flow between and out of the tanks illustrates different metabolic rates. While this model is based on the critical power concept, it introduces a time-dependent aerobic metabolic rate and utilization of anaerobic metabolic stores at power levels below critical power, thus it should not be considered a critical power model. This model enables continuous assessment of both the aerobic and anaerobic energy systems in relation to each other, as well as an arbitrarily varying power output. Similarly to Skiba et al. (2015), the recovery of the anaerobic work capacity is dependent on both the amount of expended anaerobic energy and the power output in relation to the model’s maximum aerobic rate. However, this model has not been thoroughly validated.

The Margaria-Morton model was proposed as an idea by Margaria (1976) and later mathematically described by Morton (1986), and eventually further developed by Morton (1990). This is a further progression from the critical power concept as it offers the possibility of continuous assessment of all three major metabolic pathways based on power output. This is also a hydraulic conceptualization model, where both the power output and the amount of expended energy in each metabolic system affect its metabolic rate and recovery. However, this model has not been validated. Behncke (1993) has employed an adapted form of this model to running world records of varying distances but made no further model validation. Although Sundström et al. (2014) compared the Margaria-Morton model with two other models, these models were solely evaluated with regards to previous physiological findings, using no experimental data.

Quantifying the underlying mechanisms of bioenergetics using measurements of $\dot{V}\,O_{2}$, along with a bioenergetic model, can give valuable insight into individual strengths and weaknesses. A bioenergetic model can also be used for race time predictions and optimization of pacing strategies through numerical simulations. Such specific information would be of great benefit for coaches and athletes when customizing training programs and preparing for a competition. In essence, a valid and reliable bioenergetic model with anaerobic recovery, continuously describing the utilization of both aerobic and anaerobic metabolic pathways as a function of arbitrary power output, would be a valuable tool in sports like cross-country skiing and road cycling.

Out of the models presented above, only those of Morton (1986, 2006) enable continuous assessment of both aerobic and anaerobic metabolic energy components in combination with an arbitrary varying power output. Unfortunately, both these models lack thorough experimental validation. Both models (Morton, 1986, 2006) comprise parameters that can be estimated individually, for an athlete-specific model representation. However, appropriate values of all the parameters cannot be established through standard physiological experiments and some of the model parameters do not even have a direct physiological equivalent. Thus, a method is needed that estimates these parameters for each individual using measurement data. Preferably, measurement data should include an exercise where both the aerobic and the anaerobic metabolic systems are contributing substantially to the performance, to increase the sensitivity of the dependent variables to changes in model parameter values (Kalaba and Spingarn, 1973). A suitable sport that fulfills those requirements is sprint cross-country skiing (Andersson et al., 2017).

The aim of the current study was to: (1) develop a method of individual model parameter estimation for the two suitable bioenergetic models; and (2) to assess and compare the validity and reliability of the models in their continuous prediction of aerobic and anaerobic metabolic energy utilization during sprint roller skiing with a variable power output.

## Materials and Methods

The method is divided into four sections. Section “Experimental Data”, describes the data collection and explains the mathematical preprocessing of the measured data from 11 participants performing sprint roller-skiing time trials. This experimental data was used to obtain the models’ independent variable, total metabolic rate (*MR*_*tot*_), and the models’ two dependent variables, aerobic metabolic rate (*MR*_*ae*_) and accumulated anaerobic energy expenditure (*E*_*an,acc*_). Section “Bioenergetic Models”, gives the mathematical expressions of the used models and how the modeled quantities of the dependent variables are calculated, as well as a description of the model parameters. Section “Parameter Estimation”, describes the developed method of estimating the model parameters by minimizing the difference between the measured and modeled dependent variables. Section “Statistics”, describes the statistical analyses used to determine the validity and reliability of the employed models.

### Experimental Data

Experimental data from a previous study was used, where 11 male cross-country skiers (age: 24.3 ± 3.6 years, height: 182.1 ± 5.1 cm, body mass: 78.7 ± 5.9 kg, equipment mass: 4 ± 0.1 kg, V.⁢O2m⁢a⁢x 67.5 ± 3.2 mL⋅kg^–1^⋅min^–1^), competing at a national or international level, performed submaximal exercise tests for the assessment of gross efficiency and four self-paced roller-skiing sprint time trials (STT) over undulating terrain on a motor-driven treadmill (Rodby Innovation AB, Vänge, Sweden) (Andersson et al., 2017). The study was pre-approved by the Regional Ethical Review Board of Umeå University, Umeå, Sweden (#2013-59-31M) and all participants were fully informed of its nature before providing their written consent to participate.

Pulmonary oxygen uptake ($\dot{\text{V}}\,{\text{O}}_{2}$) was monitored continuously using an ergo-spirometry system AMIS 2001 (Innovision A/S, Odense, Denmark). The raw respiratory data from the ergo-spirometry mixing-chamber system yielded 10-s values. In order to enable a higher resolution during the sprint time trials (i.e., to obtain a more realistic dynamic physiological response), raw data was interpolated second-by-second using piecewise constant interpolation for each 10-s average and smoothed using a 9-s counterbalanced moving average (i.e., using a ± 4-s time-window for smoothing) which was conducted twice. For the start-point of the sprint time trials, a gradual increase in the smoothing function time-window was used up to the fifth second whereafter the 9-s counterbalanced moving average could be used. For the end-point of the sprint time trial, the same principle was used but with a gradual decrease in the smoothing time window over the last 4 s. Thereafter, respiratory data was time interpolated to fit the treadmill data. The gas concentration data in the mixing chamber was synchronized to the corresponding breath to reduce the imposed mixed-volume delay and analyzer delay. Pulmonary $\dot{\text{V}}\,{\text{O}}_{2}$ was used for the estimation of the aerobic metabolic rate and therefore we did not compensate for the delay between pulmonary $\dot{\text{V}}\,{\text{O}}_{2}$ and muscle $\dot{\text{V}}\,{\text{O}}_{2}$.

The rolling resistance of the roller skis was assessed as described previously by Ainegren et al. (2008) and evaluated according to Andersson et al. (2017). Due to the different modes of motion in double poling and diagonal stride, rolling resistance was measured with the normal load distributed evenly between two roller skis for double poling and with the entire load on a single ski for diagonal stride.

**FIGURE 1:**
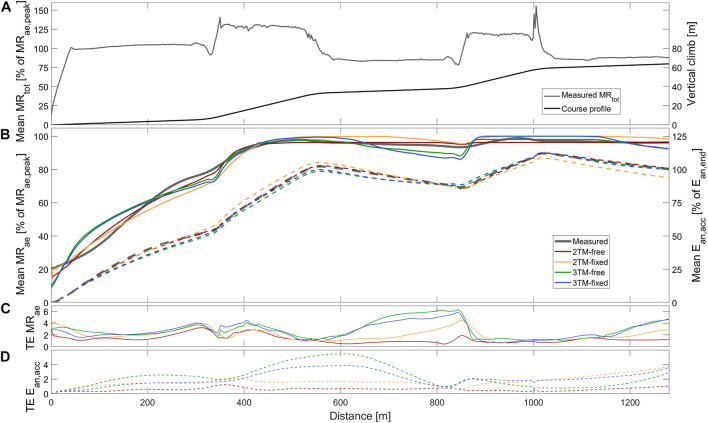
Measured and modeled metabolic variables during STT1 including **(A)** group mean of total metabolic rate (MR_tot_), course profile, **(B)** mean aerobic metabolic rate (MR_ae_, solid), accumulated anaerobic energy expenditure (E_*an*,*acc*_, dashed), **(C)** typical error of aerobic metabolic rate (TE MR_ae_), and **(D)** typical error of anaerobic accumulated energy expenditure (TE E_*an*,*acc*_). Metabolic rates are expressed relative to peak aerobic metabolic rate in STT1 (MR_*ae*,*peak*_) and accumulated anaerobic energy expenditure is expressed relative to the accumulated anaerobic energy expenditure at the end of the course (E_*an*,*end*_).

**FIGURE 2:**
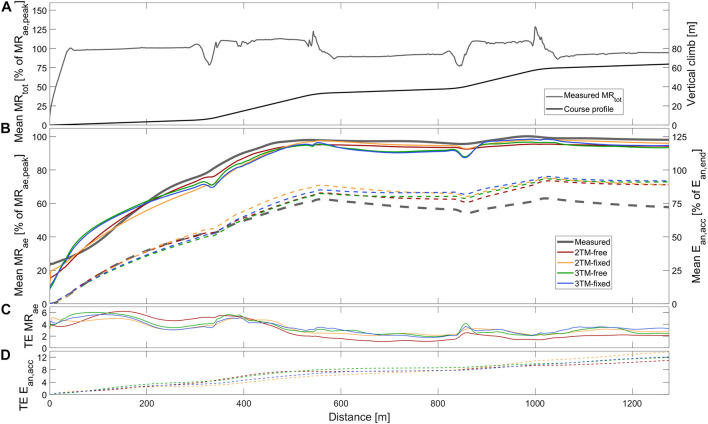
Measured and modeled metabolic variables during STT2 including **(A)** group mean of total metabolic rate (MR_tot_), course profile, **(B)** mean aerobic metabolic rate (MR_ae_, solid), accumulated anaerobic energy expenditure (E_*an*,*acc*_, dashed), **(C)** typical error of aerobic metabolic rate (TE MR_ae_), and **(D)** typical error of anaerobic accumulated energy expenditure (TE E_*an*,*acc*_). Metabolic rates are expressed relative to peak aerobic metabolic rate in STT1 (MR_*ae*,*peak*_) and accumulated anaerobic energy expenditure is expressed relative to the accumulated anaerobic energy expenditure at the end of the course (E_*an*,*end*_).

The main performance test consisted of four STTs interspersed with 45 min of recovery. However, in the current investigation, only physiological and kinematic data from the first sprint time trial (STT1) and second sprint time trial (STT2) were analyzed. The course profile consisted of three flat sections (1°) interspersed with two uphill sections (7°). The participants used double poling on the flat sections and diagonal stride on the uphill sections (see course profile in Figures 1,2). The course profile was programmed into the control unit of the motor-driven treadmill. Due to restrictions in the software of the treadmill control, the course profile differed slightly between trials. Therefore, the sprint course ranged from 1277 to 1290 m and the total climb ranged from 63.2 to 64.2 m. The skier controlled the treadmill speed by adjusting the position on the belt. A laser device detected this position and a control unit of the treadmill increased (2.45 m⋅s^–2^) or decreased (1.44 m⋅s^–2^) the speed if the skier moved to the front or rear zones of the treadmill, respectively (Swarén et al., 2013). Distance traveled was recorded every 0.406 s, resulting in *n* = 527 to 601 synced values for time, speed, and position, along the course, as well as STT finishing time.

Total metabolic rate in Watts was calculated according to the following power balance model


(1) $$M\,R_{t\,o\,t}=\frac{P}{G\,E}=\frac{m\,g\,v\,\left(\sin\alpha +\mu \,\cos\alpha \right)}{G\,E}$$

where *P* is the propulsive power output to overcome gravity and rolling resistance, *GE* is the gross efficiency, *m* is the combined mass of skier and equipment, *v* is the treadmill speed, *g* is the acceleration of gravity (9.81 m⋅s^–2^), α is the incline of the slope, and μ is the coefficient of rolling resistance. Prior to the STTs, submaximal tests were performed to evaluate the impact of speed and incline on gross efficiency in double poling and diagonal stride. Steady-state oxygen uptake ($\dot{\text{V}}\,{\text{O}}_{2}$) was measured at different speeds and inclines (for details see Andersson et al., 2017). Individual gross efficiency relationships were derived by combining steady-state power output and regression analysis of submaximal metabolic rates as functions of speed and slope, according to


(2) $$G\,E=\frac{P_{s\,u\,b}}{M\,R_{s\,u\,b}},$$

where *P*_*sub*_ is the estimated power output in the submaximal tests and *MR*_*sub*_ is the corresponding metabolic rate. *P*_*sub*_ was calculated by using the numerator in Equation (1). Moreover, the metabolic rate at submaximal steady-state exercise is primarily aerobic, and therefore calculated according to Weir (1949)


(3) $$M\,R_{s\,u\,b}=\dot{\text{V}}\,{\text{O}}_{2}\,\left(76.7\,R\,E\,R+272\right),$$

where $\dot{\text{V}}\,{\text{O}}_{2}$ is measured in L⋅min^–1^ at standard temperature and pressure, and *RER* is the measured respiratory exchange ratio. *MR*_*sub*_was independently modeled for double poling and diagonal stride as functions of speed and incline by least square regression analysis. In double poling, the regression analysis fitted second-order polynomials that were combined into the following equation


(4) $$M\,R_{D\,P}=\left(q_{1}\,v^{2}+q_{2}\,v+q_{3}\right)\,\left(q_{4}\,\alpha^{2}+q_{5}\,\alpha +q_{6}\right),$$

where *q_1-q_6* are regression coefficients. On the other hand, metabolic rate in diagonal stride was modeled by fitting linear relationships that were combined to the following equation


(5) $$M\,R_{D\,I\,A}=\left(q_{7}\,v+q_{8}\right)\,\left(q_{9}\,\alpha +q_{10}\right),$$

where *q*_7_−*q*_10_ are regression coefficients. *MR*_*DP*_ and *MR*_*DIA*_ were used as *MR*_*sub*_ in Equation (2) to calculate speed-, incline-, and sub-technique specific gross efficiencies for the STT (Andersson et al., 2017, 2020). The regression equations above are limited to model gross efficiency within, or close to, the domain tested. Therefore, special arrangements were made to avoid unrealistic efficiencies. Firstly, a modeled gross efficiency, during the STTs, that exceeded the highest measured gross efficiency from the submaximal test, was truncated to the peak value, specific for each individual and sub-technique. Secondly, subzero gross efficiency values were truncated to zero.

During the STTs, anaerobic metabolic rate was calculated as


(6) $$M\,R_{a\,n}=M\,R_{t\,o\,t}-M\,R_{a\,e}$$

where *MR*_*ae*_ is the aerobic metabolic rate calculated the same way as *MR*_*sub*_ in Equation (3) but with RER set to 1.00, i.e., assuming 100% carbohydrate utilization. The anaerobic metabolic rate was adjusted to zero from the onset of exercise up until the first time it was exceeded by the total metabolic rate. This to prevent negative anaerobic metabolic rate (e.g., anaerobic recovery) before any anaerobic energy had been used. From the anaerobic metabolic rate, the continuous accumulated anaerobic energy expenditure (*E*_*an,acc*_) was calculated as the cumulative integral of the anaerobic metabolic rate.

In the bioenergetic model parameter estimation and model validation, *MR*_*tot*_ was used as input data to the bioenergetic models (independent variable). Both *MR*_*ae*_ and *E*_*an,acc*_ were considered output data (dependent variables) and used to compare the agreement between experiments and models. Additionally, the accumulated anaerobic energy expenditure at the very end of a STT (*E*_*an,end*_) was also used as a measure of overall model validity regarding the anaerobic energy. The mitochondrial respiration is naturally rate limited and the collected anaerobic systems are naturally capacity limited (Moritani et al., 1981). Hence, we chose the aerobic metabolic *rate* and accumulated anaerobic *energy* expenditure as dependent variables for the bioenergetic model parameter estimation.

### Bioenergetic Models

The models of Morton (1986, 2006) are both based on a hydraulic tank analogy, where the energy available from different metabolic pathways is represented by fluids in separate tanks. The utilization of each metabolic pathway, represented by fluid flow, is governed by the relative fluid level difference between the tanks, the maximum flow capacity of the pipes connecting the tanks, and the fluid flow through the outlet pipe. Model parameters, such as tank volumes, maximum pipe flow capacities, and relative vertical positioning of the tanks, represent physiological characteristics of the human energetic system. By estimating these model parameters based on individual measurement data, individually representative bioenergetic models can be obtained.

**FIGURE 3:**
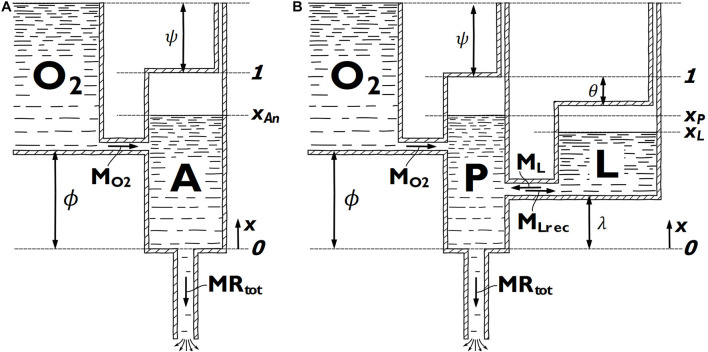
Diagrammatical representations of **(A)** the critical-power derived two-tank model (2TM) and **(B)** the adjusted Margaria-Morton three-tank model (3TM). The x-coordinate is used to express fluid surface level positions in each tank.

In the model of Morton (2006), the metabolic energy is considered to come from either aerobic energy through mitochondrial respiration, represented by the O_2_ tank, or all metabolic pathways of anaerobic energy lumped together, represented by the A tank (Figure 3A). The aerobic pathway is considered of infinite capacity, while the total anaerobic capacity (*E*_*an,max*_) is represented by the volume of the A tank. The maximum aerobic metabolic rate is denoted *M*_*O2*_ and is represented by the maximum possible flow through the pipe connecting the two tanks. The aerobic metabolic rate at any given time is represented by the concurrent flow through this pipe. The anaerobic metabolic rate at any given time is represented by the rate of change of fluid in the A tank and the total metabolic rate at any given time is represented by the flow through the outlet pipe at the bottom of the A tank. In the present study, we adjusted the model of Morton (2006) to allow a non-zero aerobic metabolic rate at rest. This is achieved by allowing the O_2_ tank to reach above the A tank. The level difference between the top of the two tanks is denoted ψ. The parameter ϕ describes the difference in tank bottom level and hence influences the response of the aerobic system. Both ϕ and ψ are considered individual parameters of the model, but they have no direct physiological analogy. The adjusted model of Morton (2006) in this study is henceforth referred to as the two-tank model (2TM).

Mathematically, the 2TM is expressed as


(7) $$A_{a\,n}\,\frac{d\,x_{A\,n}}{d\,t}=M\,R_{a\,e}^{\prime }-M\,R_{t\,o\,t}$$

(8) $$\left\{ \begin{array}{cc} M\,R_{a\,e}^{{}^{\prime }}=\frac{1+\psi -x_{A\,n}}{1+\psi -\phi }\,M_{O\,2}, & x_{A\,n}>\phi \\ M\,R_{a\,e}^{{}^{\prime }}=M_{O\,2}, & x_{A\,n}\leq \phi \end{array}\right.$$

(9) $$E_{a\,n,a\,c\,c}^{{}^{\prime }}=\left(1-x_{A\,n}\right)\,A_{a\,n}$$

where *x*_*An*_ is the fluid level in the A tank, $\frac{d\,x_{A\,n}}{d\,t}$ is the rate of change of the fluid level in the A tank, *A*_*an*_ is the cross-sectional area of the A tank and is equal to *E*_*an,max*_ since the height of the A tank is equal to one. In bioenergetic terms, the left-hand side of Equation (7) is the anaerobic metabolic rate ($A_{a\,n}\,\frac{d\,x_{A\,n}}{d\,t}=M\,R_{a\,n}$), the first term on the right-hand side is the aerobic metabolic rate, and the last term is the total metabolic rate. Equation (8) gives the aerobic metabolic rate, $M\,R_{a\,e}^{\prime }$, where ′ specifies model data (in contrast to experimental data) and Equation (9) gives the accumulated anaerobic energy expenditure, $E_{a\,n,a\,c\,c}^{\prime }$. One additional restriction is applied to the model. If the anaerobic tank is full, *x*_*An*_=1, and the metabolic rate is less than the aerobic metabolic rate at rest, $M\,R_{t\,o\,t}<\frac{\psi }{1-\psi -\phi }\,M_{O\,2}$, the aerobic metabolic rate is set equal to the total metabolic rate, $M\,R_{a\,e}^{\prime }=M\,R_{t\,o\,t}$ and the rate of change of the anaerobic energy level is set to zero, $\frac{d\,x_{A\,n}}{d\,t}=0$. In practice, this may only occur at the very beginning of a STT and only if the aerobic rate at rest is overestimated by the model.

The model of Morton (1986) is also slightly adjusted in this study and the adjusted version is referred to as the three-tank model (3TM). The 3TM considers metabolic energy from mitochondrial respiration (aerobic pathway), lactic glycolysis, and the phosphagen system, represented by the O_2_ tank, L tank, and P tank, respectively (Figure 3B). In the 3TM, as in the 2TM, the aerobic pathway is considered of infinite capacity and the maximum aerobic metabolic rate is denoted *M*_*O2*_. In the 3TM, *M*_*O2*_ is represented by the maximum possible flow through the pipe connecting the O_2_ tank and the P tank. The maximum utilization rate of the lactic glycolytic system (*M_L*) is represented by the maximum flow capacity from the L tank to the P tank, while a separate maximum flow capacity from the P tank to the L tank (*M*_*Lrec*_) represent the maximum recovery rate of the lactic glycolytic system. The utilization rate of the aerobic and the lactic glycolytic systems at any given time is represented by the concurrent flow through the pipes connecting the O_2_ tank to the P tank and the L tank to the P tank, respectively. The utilization rate of the phosphagen system at any given time is represented by the rate of change of fluid in the P tank and the total metabolic rate at any given time is represented by the flow through the outlet pipe at the bottom of the P tank. The total capacity of the phosphagen system (*E*_*P,max*_) and lactic glycolytic system (*E*_*L,max*_) is represented by the P tank and L tank volumes, respectively. The difference in tank bottom level of the O_2_ tank and L tank, relative to the bottom level of the P tank, is denoted by ϕ and λ, respectively, and they will influence the 3TM response of the respective metabolic pathway. In the same manner as for the 2TM, but in contrast to Morton (1986), the O_2_-tank in the 3TM can reach above the P tank, allowing a non-zero aerobic metabolic rate at rest. This level difference of the tank tops is decided by ψ. The difference in tank top level between the L tank and P tank is denoted θ. The parameters *ϕ*,*θ*,*λ*, and ψ will all influence the response of the various modeled metabolic pathways, but none of these parameters have any direct physiological analogy.

The 3TM model is mathematically expressed as


(10) $$A_{P}\,\frac{d\,x_{P}}{d\,t}=M\,R_{a\,e}^{\prime }-A_{L}\,\frac{d\,x_{L}}{d\,t}-M\,R_{t\,o\,t}$$

(11) $$A_{L}\,\frac{d\,x_{L}}{d\,t}=-\frac{x_{L}-x_{P}}{1-\theta -\lambda }\,M_{L}$$

(12) $$M\,R_{a\,e}^{\prime }=\frac{1+\psi -x_{P}}{1+\psi -\phi }\,M_{O\,2}$$

(13) $$E_{a\,n,a\,c\,c}^{\prime }=\left(1-x_{P}\right)\,A_{P}+\left(1-\theta -x_{L}\right)\,A_{L}$$

where *x*_*P*_ and *x_L* are the fluid levels in the P tank and L tank, respectively. The time derivatives of *x_P* and *x_L* are the rates of change of fluid levels in the respective tanks. *A_P* and *A_L* are the respective cross-sectional tank areas, where *A_P* is equal to *E*_*P,max*_, since the height of the P-tank is equal to one, and *A_L* is given by


(14) $$A_{L}=\frac{E_{L,m\,a\,x}}{1-\theta -\lambda }$$

The terms in equation (10), from left to right, are the utilization rate of the phosphagen system, the aerobic system, the lactic glycolytic system, and the total metabolic rate, respectively.

To complete the 3TM, the following restrictions, which account for specific combinations of tank levels, need to be applied


(15) $$\begin{array}{cc} x_{P}>x_{L}: & M_{L}=M_{L\,r\,e\,c} \end{array}$$

(16) $$\begin{array}{cc} x_{P}<\phi : & M\,R_{a\,e}^{\prime }=M_{O\,2} \end{array}$$

(17) $$x_{P}<\lambda :A_{L}\,\frac{d\,x_{L}}{d\,t}=-\frac{x_{L}-\lambda }{1-\theta -\lambda }\,M_{L}$$

(18) $$\left\{ \begin{array}{cc} \begin{array}{c} x_{L}=1-\theta \\ x_{P}>x_{L} \end{array}: & \frac{d\,x_{L}}{d\,t}=0 \end{array}\right.$$

(19) $$\left\{ \begin{array}{cc} \begin{array}{c} x_{P}=1\\ M\,R_{t\,o\,t}<\frac{\psi }{1-\psi -\phi }\,M_{O\,2} \end{array}: & \left\{ \begin{array}{c} M\,R_{a\,e}^{\prime }=M\,R_{t\,o\,t}\\ \frac{d\,x_{P}}{d\,t}=0\\ \frac{d\,x_{L}}{d\,t}=0 \end{array}\right. \end{array}\right.$$

where Equation (15) ensures the recovery rate of the lactic glycolytic system is restricted to a maximum of *M*_*Lrec*_, Equation (16) ensures the aerobic metabolic rate to not exceed *M*_*O2*_, Equation (17) ensures the lactic glycolysis rate to not exceed *M_L* and Equation (18) prevents overfilling of the L tank. Equation (19) sets the aerobic metabolic rate equal to the total metabolic rate and negates any change in the anaerobic systems if the total metabolic rate is lower than the model’s aerobic metabolic rate at rest.

The 2TM and 3TM include four and nine parameters, respectively, that need to be determined from experimental data or known physiological quantities, to obtain an individually representative bioenergetic model. Previously published data on the participants of the current study indicate, that $\dot{V}\,O_{2,m\,a\,x}$ derived from an incremental $\dot{V}\,O_{2,m\,a\,x}$ test was similar to peak $\dot{V}\,O_{2}$ obtained during the STTs (Andersson et al., 2017). Hence, it is desirable that the maximum aerobic rate of the models, *M*_*O2*_, attains a value close to the energy equivalent of $\dot{V}\,O_{2,m\,a\,x}$ and therefore two versions of both the 2TM and 3TM were used. These versions either had *M*_*O2*_ fixed to the measured peak energy equivalent of $\dot{V}\,O_{2}$ (*MR*_*ae,peak*_) from STT1 (2TM-fixed and 3TM-fixed) or *M*_*O2*_ free to be determined by the parameter estimation (2TM-free and 3TM-free). Apart from *M*_*O2*_ in the cases when it was fixed, all parameters were decided by the parameter estimation with data from STT1 for each individual.

### Parameter Estimation

All bioenergetic models were programmed in MATLAB (R2020b, Mathworks Inc., Natick, MA, United States) and fitted to each individual through parameter estimation, by minimizing the mean squared error (MSE) between model prediction and measurements for both *MR*_*ae*_ and *E*_*an,acc*_. This was done using the non-linear gray-box parameter estimation solver, *nlgreyest*, in the System identification toolbox in MATLAB (R2020b, Mathworks Inc., Natick, MA, United States). A least-square non-linear search method (*lsqnonlin*) was used, with a cost function tolerance of 10^–6^, a step tolerance of 10^–5^, and a maximum number of iterations set to 100. The Trust region reflective optimization algorithm in MATLAB was used. The cost function for the *lsqnonlin* solver to be minimized was defined as the weighted sum of the MSE between the model and measured data of the two dependent variables. The MSE of *MR*_*ae*_ was given the weight one, while the weight of MSE of *E*_*an,acc*_ for each individual was calculated as


(20) $$w={\left(\frac{{\bar{M\,R}}_{a\,e}}{{\bar{E}}_{a\,n,a\,c\,c}}\right)}^{2}$$

where ${\bar{M\,R}}_{a\,e}$ and ${\bar{E}}_{a\,n,a\,c\,c}$ are the measured mean in STT1 of the aerobic metabolic rate and accumulated anaerobic energy expenditure, respectively. The lowest obtained value of the root of the cost function was used as criteria for selecting the optimal set of estimated parameters.

To ensure a global optimum, a multi-start method was applied, so that the parameter estimation for each individual and each model was run with various combinations of initial parameter values. Moreover, to avoid unrealistic solutions, the range of allowed parameter values was restricted by lower and upper bounds. For 2TM-free and 3TM-free, where *M*_*O2*_ was allowed to vary, we used a lower and upper bound of, respectively, 90 and 110% of the peak aerobic metabolic rate (*MR*_*ae*,*peak*_), corresponding to the peak oxygen uptake ($\dot{V}\,O_{2,p\,e\,a\,k}$) for each individual reached in STT1. 100% of *MR*_*ae*,*peak*_ was used as the initial condition.

The rate of lactic glycolysis has been reported to be close to double that of the aerobic metabolic rate (Baker et al., 2010). Therefore 160 and 240% of *MR*_*ae*,*peak*_ was used as lower and upper bounds of *M_L*, respectively, and 180, 200, and 220% of *MR*_*ae*,*peak*_ were used as initial values for 3TM-free and 3TM-fixed.

The maximum measured accumulated anaerobic energy expenditure, *E*_*an,peak*_, from STT1 for each individual was used as an approximation of each individual’s maximum anaerobic capacity. The human anaerobic capacity has been reported to be 26% phosphagen system and 74% lactic glycolytic system (Sahlin, 2014). Adding the additional capacity available through the oxygen stored in muscles and blood to the phosphagen system (since it is neither lactic-acid forming nor dependent on the respiratory system), the distribution will be 33/67% (Medbø et al., 1988). Based on this, the lower and upper bounds for *E*_*L,max*_ was set to 60 and 80% of *E*_*an,peak*_ and for *E*_*P,max*_ to 20 and 40% of *E*_*an,peak*_. Initial values were set to a distribution of 75/25 or 65/35% of *E*_*an,peak*_ between *E*_*L,max*_ and *E*_*P,max*_. For the 2TM-free and 2TM-fixed, lower and upper bounds for *E*_*an,max*_ was set to 80 and 120% of *E*_*an,max*_ and 90, 100, and 110% were used as initial values.

Muscle lactate levels from muscle biopsies at rest, at the cessation of intense exercise, and after 3.4 and 10.8 min of rest have been reported to be 1.7, 22.6, 13.4, and 3.2 mmol⋅(kg wet mass)^–1^, respectively, (Bangsbo et al., 1994). Assuming the body strives to restore at-rest lactate levels, curve fitting a second degree polynomial of this data yields a lactate recovery rate at the cessation of exercise of 3.07 mmol⋅(kg wet mass)^–1^⋅min^–1^. This corresponds to a reduction of 0.24% of maximal accumulated lactate per second. Assuming a maximum of 80% of the anaerobic energy coming from the lactic glycolytic system, the initial values and bounds for *M*_*Lrec*_, were all related to an approximated maximum recovery rate of the lactic glycolytic system of 0.24%⋅80%⋅*E*_*an*,*max*_. The upper and lower bounds were specified as 50 and 150% of this value and initial values were 80, 100, and 120% thereof.

The parameters ϕ, θ, and λ all have geometrically given lower and upper bounds of zero and one, respectively (Figure 3), and these parameters were not further restricted. The additional geometrical requirement that *θ* + *λ*≤1 was not actively implemented, but the results were checked not to violate this restriction. Preliminary parameter estimations indicated these three parameters would rarely assume values above 0.5 for 3TM-free or 3TM-fixed. Hence, 0.1 and 0.3 were used as initial values for these three parameters for the 3TM. For 2TM-free and 2TM-fixed initial values of 0.1, 0.3, and 0.5 were used for ϕ, since higher values were indicated in the preliminary parameter estimations.

Only a lower bound was applied to ψ, since preliminary parameter estimations indicated values close to 0.1 with no unrealistically large exceptions. For the 2TM-free and 2TM-fixed, 0 and 0.1 were used as initial values. For the 3TM-free and 3TM-fixed only 0.1 was used to restrict the parameter estimation solver time.

All combinations of initial parameter values were used in the multi-start parameter estimation. This gave a total of 144 combinations of initial parameter values to be run for each individual in the three-tank models (3TMs) (both fixed and free) and a total of 18 combinations for each individual in the two-tank models (2TMs) (both fixed and free).

A parameter value equal or close to any of their respective bounds could indicate that a global optimum exists with parameter values outside of the allowed range. To investigate if this was the case, a check was made for the parameters with a direct physiological analogy (*M*_*O*2_,*M*_*L*_,*M*_*Lrec*_,*E*_*P*,*max*_,*E*_*L*,*max*_,*E*_*an*,*max*_) according to


(21) $$p_{c\,h\,e\,c\,k}=\frac{p_{e\,s\,t}-p_{m\,i\,d}}{p_{m\,a\,x}-p_{m\,i\,d}}$$

where *p*_*est*_ is the parameter value from the parameter estimation and *p*_*mid*_ is the mid-range parameter value calculated as


(22) $$p_{m\,i\,d}=\frac{p_{m\,a\,x}+p_{m\,i\,n}}{2}$$

where *p*_*min*_ is the lower bound and *p*_*max*_ is the upper bound. A *p*_*check*_ value of ±1 means the parameter value is equal to the lower and upper bounds, respectively. For the remaining parameters (*ϕ*,*θ*,*λ*,*ψ*), active bounds were considered acceptable and parameters outside their respective allowed range would result in unfeasible solutions. Also, these parameters range in value from zero to one (apart from ψ having no upper bound), so the parameter values themselves indicate if any bounds were active or not. Hence, no *p*_*check*_ values were calculated for these parameters.

### Statistics

The Statistical Package for the Social Sciences (SPSS 25, IMB Corp, Armonk, NY, United States) and MATLAB R2020b (Mathworks Inc., Natick, MA, United States) were used for the statistical analyses and the level of significance was set to α = 0.05. Data were checked for normality by visual inspection of the Q-Q plots and histograms as well as using the Shapiro-Wilks test. Parametric tests were used for normally distributed data, whereas non-parametric alternative tests were used for data that mainly were non-normally distributed. Normally distributed data are presented as mean ± SD, whereas non-normally distributed data are presented as median and interquartile range (IQR). The mean difference ± 95% limits of agreement for the comparison of each bioenergetic model vs. experimental data (i.e., the measured values) were evaluated using Bland-Altman calculations (Bland and Altman, 1999). Bland-Altman calculations were performed on the average *MR*_*ae*_ and *E*_*an,end*_ from each respective sprint time trial. The mean difference was tested with a paired-sample *t*-test. In addition, the reliability of the models was evaluated via the absolute typical error for the separate pair-wise comparisons (i.e., the SD of the pair-wise differences divided by the square root of two). The instantaneous typical error in *MR*_*ae*_ and *E*_*an,acc*_, based on meter-by-meter data, was calculated for each model during the respective STT. The root mean square error (RMSE) between model simulation output and measured values, for both dependent variables, i.e., *MR*_*ae*_ and *E*_*an,acc*_, was calculated on instantaneous STT data (2.46 Hz). Both dependent variables were expressed in native units (W and J, respectively) and as a percentage of their time-mean value for each participant’s STT. A Friedman test was used to analyze the main effect of model simulation RMSE values of both *MR*_*ae*_ and *E*_*an,acc*_ for each specific STT as well as to analyze the parameter estimation solver time for the four bioenergetic models. In case of a significant main effect, *post-hoc* Wilcoxon *signed-rank* tests were used to identify specific differences between model estimates. Within-model RMSE comparisons between the two STTs were also performed by using a Wilcoxon *signed-rank* test. In addition, a Wilcoxon *signed-rank* test was used for the comparison of the model parameters between the 2TM-free and 2TM-fixed and between the 3TM-free and 3TM-fixed, respectively. For the Wilcoxon *signed-rank* tests, Bonferroni α corrections were applied in case of multiple comparisons, i.e., multiplying the *P*-value with the number of pairwise comparisons for each variable. The Bland-Altman, typical error, and RMSE calculations were done for both STT1, quantifying the validity and reliability of the parameter estimation method, and for STT2, quantifying the validity and reliability of each individually adapted bioenergetic model.

## Results

The total skiing time of STT1 was 229.2 ± 8.7 s, with a skied distance of 1285.6 ± 2.4 m, an average *MR*_*tot*_ of 1.85 ± 0.21 kW, an average *MR*_*ae*_ of 1.54 ± 0.15 kW, and *E*_*an,end*_ being 70.4 ± 27.3 kJ. For STT2 the corresponding measured quantities were 232.5 ± 9.5 s, 1286.4 ± 3.6 m, 1.77 ± 0.14 kW, 1.56 ± 0.14 kW, and 47.9 ± 20.0 kJ, respectively. The measured instantaneous average *MR*_*ae*_ is, together with the course profile, shown in Figures 1A, 2A for STT1 and STT2, respectively. *MR*_*tot*_ is shown to drop below *MR*_*ae*_ during the second and third flat sections of both STTs (Figures 1,2), allowing anaerobic recovery according to the bioenergetic model formulations. The interpolation coefficients from the regression analyses used to calculate *MR*_*DP*_ and *MR*_*DIA*_ are given in Table 1.

**TABLE 1:** Regression coefficients of the gross efficiency relationships derived from submaximal steady state exercise (Equations 4 and 5).

**Coefficient**	**q_1_**	**q_2_**	**q_3_**	**q_4_**	**q_5_**	**q_6_**	**q_7_**	**q_8_**	**q_9_**	**q_10_**
Mean	0.00281	0.00266	0.0595	–0.0374	0.1709	0.866	586	–29.4	0.0341	0.762
SD	0.00223	0.0209	0.0403	0.0410	0.1658	0.1251	67.7	127.9	0.00799	0.0559

*No statistical comparisons were made for the gross efficiency coefficients.*

### Model Validity and Reliability

**FIGURE 4:**
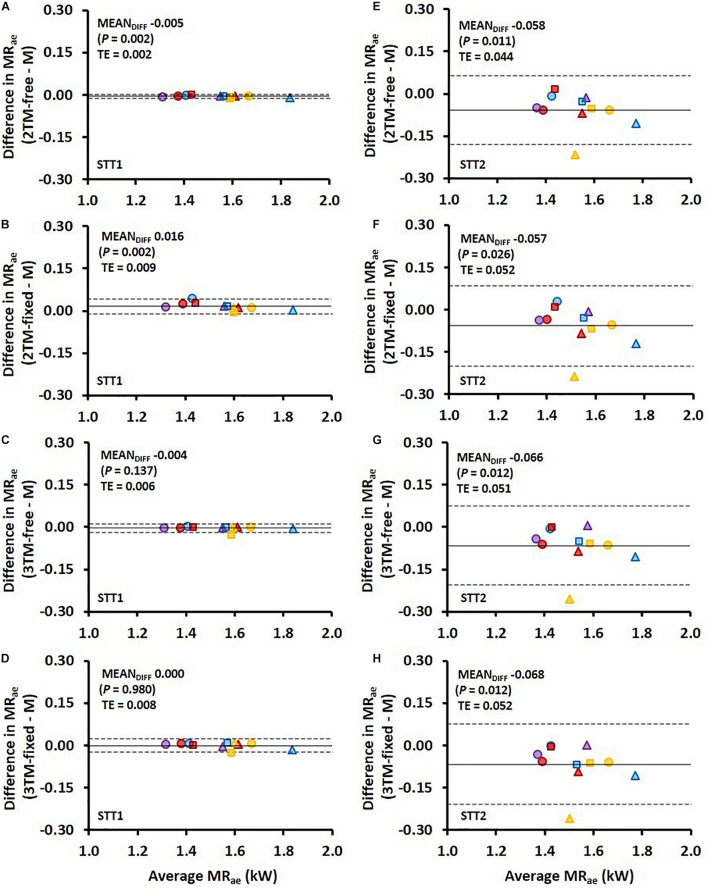
Bland-Altman plots representing the mean difference (MEAN_*DIFF*_) ± 95% limits of agreement (i.e., 1.96 SD) in the average aerobic metabolic rate (MR_*ae*_) associated with the first sprint time trial (STT1) presented in **(A–D)** and for the second sprint time trial (STT2) presented in **(E–H)** for the four various bioenergetic models vs. measurement data (M). TE, typical error.

**FIGURE 5:**
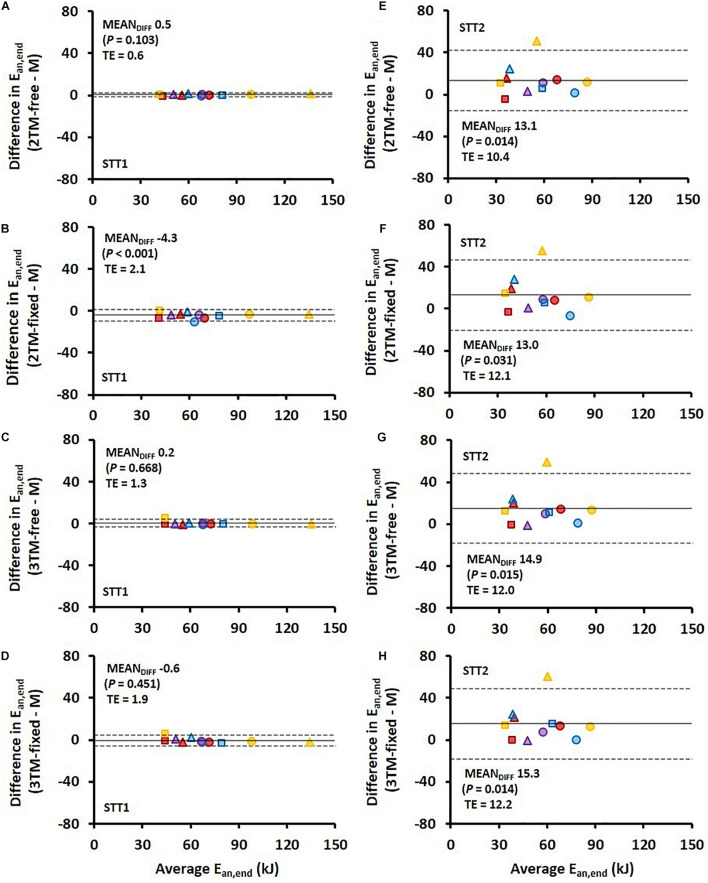
Bland-Altman plots representing the mean difference (MEAN_*DIFF*_) ± 95% limits of agreement (i.e., 1.96 SD) in the accumulated anaerobic energy contribution (E_*an*__,end_) associated with the first sprint time trial (STT1) presented in **(A–D)** and for the second sprint time trial (STT2) presented in **(E–H)** for the four various bioenergetic models vs. measurement data (M). TE, typical error.

Comparisons of *MR*_*ae*_ and *E*_*an,end*_ for each respective bioenergetics model vs. measurement data are shown for STT1 and STT2 in Figures 4,5. The model-to-measurement mean difference and typical errors in *MR*_*ae*_ were small for STT1 (see Figures 4A–D) but noticeably larger for STT2 (see Figures 4E–H). Also, the model-to-measurement mean difference and typical errors in *E*_*an,end*_ were small for STT1 (see Figures 5A–D) but noticeably larger for STT2 (see Figures 5E–H). Moreover, the model-to-measurement mean difference typical error was lower in 2TM-free compared to the other models in both *MR*_*ae*_ and *E*_*an,acc*_ for STT1 and STT2.

As shown in Figure 1B, the mean *MR*_*ae*_ for the four models vary between over and underprediction vs. measurement data along with the traveled distance in STT1, with the most pronounced over and underpredictions in the 3TM-free and 3TM-fixed models. As shown in Figure 2B, the mean *MR*_*ae*_ was underpredicted for the majority of STT2 for all models. Both the 2TM-free and 2TM-fixed models showed mean *MR*_*ae*_ to level out after ∼400 m of STT1 at a value that was equal to *M*_*O2*_ (Figure 1B). The same effect was visible also for the 3TM-free and 3TM-fixed, but with a shorter duration of the plateau. The instantaneous typical error in *MR*_*ae*_ for each respective model ranged between 1 and 6% in both STTs (see Figures 1C, 2C). However, the typical error curves differed slightly between models and the two STTs. The mean *E*_*an,acc*_ showed excellent agreement with measured data in STT1 with the typical error being less than 3% of *E*_*an,end*_ during the majority of the STT (see Figure 1D). On the contrary to STT1, all four models showed a growing over-prediction in *E*_*an,acc*_ throughout STT2, which also was accompanied by a continuously growing typical error, indicating large between-participant variation (see Figures 2B,D).

As shown in Table 2, the RMSE of both *MR*_*ae*_ and *E*_*an,acc*_ in absolute terms for both STT1 and STT2 were the lowest for the 2TM-free and the highest for the 3TM-fixed. Compared with the 2TM-free in STT1, the median values of RMSE of *MR*_*ae*_ were 57, 48, and 96% higher for the 2TM-fixed, 3TM-free, and 3TM-fixed, respectively. For the RMSE of *E*_*an,acc*_ the same numbers were 256, 53, and 119%, respectively. Compared with the 2TM-free in STT2, the median values of RMSE of *MR*_*ae*_ were 15, 29, and 37% higher for the 2TM-free, 3TM-free, and 3TM-fixed, respectively. The RMSE of *MR*_*ae*_ as percentage of ${\bar{M\,R}}_{a\,e}$ was larger for all four models compared to the RMSE of *E*_*an,exp*_ as a percentage of ${\bar{E}}_{a\,n,e\,x\,p}$ for STT1, while the opposite was true for STT2 (Table 2).

**TABLE 2:** The median and interquartile range (IQR) of root mean square error (RMSE) for the aerobic metabolic rate and accumulated anaerobic energy expenditure in absolute and relative numbers for STT1 and STT2. Also reported is the weighted RMSE of *MR*_*ae*_ and *E*_*an,acc*_ as described in section “Parameter Estimation”.

**STT 1**	**2TM-free**	**2TM-fixed**	**3TM-free**	**3TM-fixed**	**Main effect**
Weighted *MR*_*ae*_ and *E*_*an,acc*_	53.7 (49.2–58.9)*^*b.c.d*^*	102.0 (84.6–130.4)	77.9 (65.0–167.5)	104.1 (82.2–135.9)	*P* < 0.001
*MR*_*ae*_ [W]	50.0 (45.3–55.5)*^*b.c.d*^*	78.6 (69.0–91.7)	74.0 (63.0–135.6)	97.8 (75.3–122.9)	*P* < 0.001
*E*_*an,acc*_ [kJ]	0.61 (0.54–0.74)*^*b.c.d*^*	2.15 (1.43–2.87)	0.93 (0.73–2.77)	1.33 (1.08–1.91)	*P* < 0.001
*MR*_*ae*_ [% of mean]	3.4% (2.7–3.6%)*^*b.c.d*^*	5.7% (4.2–6.5%)	5.3% (4.1–8.7%)	6.1% (5.4–7.9%)	*P* < 0.001
*E*_*an,acc*_ [% of mean]	1.3% (0.9–1.5%)*^*b.c.d*^*	3.8% (2.5–6.3%)	1.8% (1.4–6.3%)	2.6% (2.1–3.7%)	*P* < 0.001
**STT2**					
*MR*_*ae*_ [W]	77.6 (70.4–96.8)*^*c.d;*^* ^$^	88.9 (58.7–113.9)	100.1 (88.7–145.8)	106.1 (89.6–135.8)	*P* < 0.001
*E*_*an,acc*_ [kJ]	5.12 (3.64–9.24)^$^	7.33 (3.46–11.91)	6.30 (4.01–11.06)^$^	8.96 (2.19–11.37)^$^	*P* = 0.664
*MR*_*ae*_ [% of mean]	5.1% (4.6–6.8%)*^*c.d;*^*	5.8% (4.1–7.2%)	6.5% (5.7–8.6%)	7.5% (5.7–8.3%)	*P* < 0.001
*E*_*an,acc*_ [% of mean]	11.7% (8.6–30.9%)	14.1% (6.8–39.8%)	14.4% (8.5–33.1%)	17.2% (5.1–34.1%)	*P* = 0.664

*STT1, the first sprint time trial; STT2, the second sprint time trial; MR*_*ae*_, *aerobic metabolic rate; E*_*an,acc*_, *accumulated anaerobic energy expenditure.*

*P-values for the main effect of model on the physiological variables in each sprint time trial were obtained by a Friedman test. Pair-wise post-hoc comparisons were conducted with a Wilcoxon signed-rank test and applying a Bonferroni α correction for multiple comparisons. ^*b*^Statistically significantly different from 2TM-fixed (P < 0.05). ^*c*^Statistically significantly different from 3TM-free (P < 0.05). ^*d*^Statistically significantly different from 3TM-fixed (P < 0.05). ^$^P = 0.013 for STT1* vs. *STT2 (assessed with a Wilcoxon signed-rank test).*

### Estimated Parameters

The estimated parameters values for all four models are shown in Table 3. For the 2TM-free and 2TM-fixed, all estimated parameters were found to be significantly different, with a difference in median values of 4, 8, −24, and 28% for *M*_*O*2_,*E*_*an*,*max*_,*ϕ*, and ψ, respectively, for the 2TM-fixed compared to 2TM-free. The only significant differences in estimated parameter values between the 3TM-free and 3TM-fixed were found in *M*_*O*2_,*E*_*P*,*max*_, and ϕ, with *M*_*O2*_ and *E*_*P,max*_ being 0.7 and 0.3% larger in 3TM-fixed compared to 3TM-free and ϕ being 25% smaller.

**TABLE 3:** The median and interquartile range (IQR) of estimated optimal parameters for the four bioenergetic models.

**Parameters**	**2TM-free**	**2TM-fixed**		**3TM-free**	**3TM-fixed**	
*M*_*O2*_ [kW]	1.75 (1.55–1.85)	1.82 (1.65–1.92)	*P* = 0.003	1.81 (1.56–1.90)	1.82 (1.65–1.92)	*P* = 0.026
*M_L* [kW]				4.22 (3.97–4.61)	4.38 (2.87–4.61)	*P* = 0.424
*M*_*Lrec*_ [kW]				0.17 (0.15–0.22)	0.18 (0.16–0.22)	*P* = 0.594
*E*_*P,max*_ [kJ]				30.38 (22.12–33.61)	30.46 (24.13–33.61)	*P* = 0.016
*E*_*L,max*_ [kJ]				47.59 (37.76–52.71)	48.78 (37.01–60.93)	*P* = 0.859
ϕ	0.396 (0.30–0.439)	0.302 (0.254–0.360)	*P* = 0.006	0.333 (0.290–0.404)	0.251 (0.198–0.328)	*P* = 0.004
λ				0.024 (0.014–0.129)	0.019 (0.000–0.094)	*P* = 0.075
θ				0.084 (0.075–0.086)	0.083 (0.052–0.086)	*P* = 0.286
ψ	0.126 (0.091–0.145)	0.160 (0.150–0.192)	*P* = 0.003	0.075 (0.046–0.095)	0.079 (0.051–0.135)	*P* = 0.091
*E*_*an,max*_ [kJ]	76.86 (63.77–85.40)	82.95 (68.20–93.55)	*P* = 0.003			

*M_O2_, maximum aerobic metabolic rate; M_L_, maximum lactic glycolysis rate; M_Lrec_, maximum recovery rate of lactic glycolytic system; E_P,max_, maximum capacity of the phosphagen system; E_L,max_, maximum capacity of the lactic glycolytic system; E_an,max_, maximum anaerobic capacity of the 2TMs; ϕ,λ,θ,ψ, model parameters.*

*P-values are reported for the separate pairwise comparisons of the two three-tank models (i.e., 3TM-free and 3TM-fixed) and the two two-tank models (i.e., 2TM-free and 2TM-fixed) by using a Wilcoxon signed-rank test.*

The relative contribution from the phosphagen system and the lactic glycolytic system to the total anaerobic capacity was 38.6% (35.6–39.0%) and 61.4% (61.0–64.4%), respectively, for the 3TM-free and 38.7% (37.3–39.1%) and 61.3% (60.9–62.7%), respectively, for the 3TM-fixed. The maximum glycolytic rate, *M_L*, as a percentage of *M*_*O2*_ was 244% (236–250%) and 240% (160–240%) for the 3TM-free and 3TM-fixed, respectively.

The absolute of the *p*_*check*_ values did not exceed 0.8 for any of the parameters in either the 2TM-free or 2TM-fixed. Hence, all parameter values were well within the allowed range and no global optimum of the parameter estimation was expected to be found with parameter values outside the prescribed allowed range.

The *p*_*check*_ value of *M*_*L*_,*M*_*Lrec*_, and *E*_*P,max*_ was equal to one for 8, 5, and 6 participants, respectively, for the 3TM-free and 6, 9, and 11 participants, respectively, for the 3TM-fixed. For the 3TM-fixed, a value of minus one was attained for *M_L* and *M*_*Lrec*_ for 3 and 2 participants, respectively. Also, the *p*_*check*_ value of *E*_*L,max*_ was equal to one for one participant and less than −0.9 for another participant in 3TM-fixed. The absolute *p*_*check*_ values for *M*_*O2*_ was less than 0.6 for all individuals in 3TM-free.

### Parameter Estimation Solver Times

The solver time of the parameter estimations was more than 20 times longer for 3TM-free and 3TM-fixed compared to 2TM-free and 2TM-fixed, respectively (Table 4). Furthermore, the solver time was 27% less for 3TM-fixed compared to 3TM-free and 24% less for 2TM-fixed compared to 2TM-free, though the only difference is one parameter less to estimate in each case, i.e., in the “fixed” vs. “free” models.

**TABLE 4:** The parameter estimation solver times along with descriptive data of the four bioenergetic models.

	**2TM-free**	**2TM-fixed**	**3TM-free**	**3TM-fixed**	**Main effect**
Regression time [min]	2.6 (2.1–3.4)^*b,c,d*^	2.6 (2.1–3.4)^*c,d*^	67.1 (47.4–93.8)^*d*^	49.0 (31.0–62.9)	*P* < 0.001
No. of parameters	4	3	9	8	*−*
No. of initial value combinations	18	18	144	144	*−*
No. of differential equations	1	1	2	2	

*Statistical comparisons were performed for median and interquartile range (IQR) model-specific regression times. The *P*-value was obtained by a Friedman test. Pair-wise post-hoc comparisons were conducted with a Wilcoxon signed-rank test and applying a Bonferroni correction for multiple comparisons.*

*^*b*^Significantly different from 2TM-fixed (*P* < 0.05). ^*c*^Significantly different from 3TM-free (*P* < 0.05). ^*d*^Significantly different from 3TM-fixed (*P* < 0.05). Pairwise comparisons were performed with Wilcoxon signed-rank tests.*

## Discussion

Among the investigated bioenergetic models, the 2TM-free provided the highest validity and reliability in *MR*_*ae*_ and *E*_*an,acc*_, for both the parameter estimation in STT1 and the validity and reliability evaluation in the succeeding STT2, where the individually estimated parameters were applied (for details see Table 2). However, all models struggled to capture *MR*_*ae*_ and *E*_*an,acc*_ instantaneously. During the initial rapid rise in *MR*_*ae*_, all individually optimized models altered between over and under predictions (Figures 1B, 2B and Supplementary Material). The model-to-measurement discrepancy was also apparent after the initial rapid rise in *MR*_*ae*_. In this phase of the trials, the range of *MR*_*ae*_ variation was measured to ∼100 W, which also makes the accuracy of the 2TM-free questionable, since its RMSE of *MR*_*ae*_ was ∼50 W. The poor accuracy after the initial rise adheres to the models’ inability to capture the *MR*_*ae*_ variations. This inability resulted in a plateau value slightly below *MR*_*ae,peak*_ being the best possible fit.

The modeled *MR*_*ae*_ is linearly dependent on the available anaerobic energy store in the 2TMs and phosphagen energy store in the 3TMs, respectively. At the onset of exercise, the rate of change in these energy levels is entirely dependent on *MR*_*tot*_, ψ, as well as *E*_*an,max*_ and *E*_*P,max*_, in the 2TMs and 3TMs, respectively (Figure 3). With *MR*_*tot*_ obtained from measurements and ψ mainly deciding the initial *MR*_*ae*_, the initial rate of change of *MR*_*ae*_ is mainly decided by *E*_*an,max*_ and *E*_*P,max*_, in the 2TMs and 3TMs, respectively. *E*_*an,max*_ and *E*_*P,max*_ thus governs the kinetic response of *MR*_*ae*_ and corresponds to the time constant of $\dot{\text{V}}\,{\text{O}}_{2}$ kinetics, τ (Poole and Jones, 2012). However, the kinetic response of *MR*_*ae*_ in the 2TMs and 3TMs is modeled differently to common $\dot{\text{V}}\,{\text{O}}_{2}$-kinetics models (Poole and Jones, 2012). In the 2TMs and 3TMs, rate of change of *MR*_*ae*_ is initially proportional to the accumulated anaerobic and phosphagen energy expenditures, respectively, while the rate of change of $\dot{\text{V}}\,{\text{O}}_{2}$ is commonly modeled as proportional to the difference between $\dot{\text{V}}\,{\text{O}}_{2}$ demand and the current $\dot{\text{V}}\,{\text{O}}_{2}$ (i.e., error signal). Therefore, the error signal that controls *MR*_*ae*_ in the 2TMs and 3TMs is approximately equivalent to the time integral of the error signal used in common $\dot{\text{V}}\,{\text{O}}_{2}$ kinetics models (Poole and Jones, 2012). In the simulation of all models in the current study, the initial rate of change of *MR*_*ae*_ is overestimated when compared to measurements. From observation of the simulation results, it can be seen that this overestimation is larger in the 3TMs compared to 2TMs (Figure 1B). It is reasonable to assume that this is due to *E*_*an,max*_ in the 2TMs being greater than *E*_*P,max*_ in the 3TMs, because this is analogous to a slower kinetic response in *MR*_*ae*_ in the 2TMs compared to the 3TMs. In many participants, the overestimated rate of change of *MR*_*ae*_ at the onset of exercise was followed by a corresponding phase of underestimation followed by another phase of overestimation. This finding suggests that the agreement between model and measurements is poor in the initial phase of exercise. Therefore, we conclude that the assumed linear relationship between *MR*_*ae*_ and *x*_*An*_ or *x_P* is not an accurate description of the studied skiers’ bioenergetics in the initial phase of exercise. A possible reformulation may therefore be to model the rate of change of *MR*_*ae*_ as linearly dependent on the difference between *MR*_*tot*_ and *MR*_*ae*_ in resemblance with common $\dot{V}\,O_{2}$-kinetics modeling (Poole and Jones, 2012). This may also include the initial cardiodynamic component of $\dot{V}\,O_{2}$ kinetics or a time delay in the primary component, which both were neglected in the current study. Furthermore, by compensating for the time delay between pulmonary $\dot{V}\,O_{2}$ and muscle $\dot{V}\,O_{2}$, results might better describe the time course of bioenergetic processes in the muscles. This delay is maximal at the onset of exercise after which it gradually decreases as the cardiac output increases. Although this would complicate the model by introducing more parameters, compensating for delay between pulmonary $\dot{V}\,O_{2}$ and muscle $\dot{V}\,O_{2}$ might improve the agreement in the initial phase of exercise.

The initial phase of the STTs, with a rapid rise in *MR*_*ae*_, lasted approximately a third of the total STT duration, while during the remainder of the STT, *MR*_*ae*_ was steadier. Since the data sampling frequency was constant, and the parameter estimation minimized the weighted mean squared error over all data samples, the time distribution (∼1/3 rise of *MR*_*ae*_, ∼2/3 steady *MR*_*ae*_) is an indication of the relative importance of these phases to the parameter estimation. Therefore, during longer-duration time trials, the initial phase of *MR*_*ae*_ rise would be of relatively lower importance to the parameter estimation. This could possibly result in a model with a better ability to capture the small variations during the latter phase of a time trial, but with larger errors in the initial phase. With this in mind, estimated model parameters may be specific to certain conditions of the input data and may not apply to different exercise durations or types of exercise. Further investigations are needed to determine the generalizability of the 2TMs and 3TMs.

The RMSE of *E*_*an,acc*_ as percentage of ${\bar{E}}_{a\,n,a\,c\,c}$ showed both higher validity and reliability for all four models compared to that of *MR*_*ae*_ for STT1, but the opposite for STT2. Figure 5 reveal large inter-individual differences in STT2 in all four model predictions of *E*_*an,end*_. While the model predictions were close to the target for several participants, the overall trend was an overestimation of *E*_*an,end*_. One likely contributing cause is that the participants performed STT1 at slightly different individual intensities, which probably affected their level of recovery at the start of STT2. The majority of the participants may have not fully recovered at the start of STT2, though the models were set to start with fully recovered anaerobic stores. This overestimation of initial anaerobic stores lead to a slower rise in *MR*_*ae*_ and resulted in *MR*_*ae*_ being underpredicted as is shown in Figure 4. The higher measured mean *MR*_*ae*_ at the onset of exercise in STT2 compared to STT1, further supports the observation of unrecovered anaerobic stores in STT2.

Another possible source of error is related to the regression models used for determining the supramaximal gross efficiency during the STTs as based on the experimental data. This ineluctable problem can be related to the uncertainty when using a speed (or power output) vs. metabolic rate relationship determined at submaximal exercise intensities and using extrapolation for determining the required metabolic rate (and/or gross efficiency) at higher exercise intensities. This problem might be specifically problematic when using polynomial regression models due to overfitting. However, the curvilinear data observed for double poling (see Equation 2) made it impossible to use the same linear models that were used for diagonal stride (for details see Andersson et al., 2020). This potential problem, which is likely to be higher for double poling than for diagonal stride, helps to explain some of the unreasonable values of gross efficiency that were estimated and thus truncated during the transitions between the two sub-techniques (for details see methods section “Experimental Data”). Since the modeled supramaximal gross efficiency during the STTs was used to calculate *MR*_*tot*_ and that *MR*_*tot*_ was used as input data to the bioenergetic models, it is possible that the modeled supramaximal gross efficiency during the STTs could be a potential source of error that contributed to the variation in the predicted *E*_*an,acc*_ between bioenergetic models and STTs.

The RMSE of *E*_*an,acc*_ was shown to increase more between the 2TM-free and 2TM-fixed than between the 3TM-free and 3TM-fixed. This could partly reflect the stiffness of the 2TMs compared to the 3TMs. The 2TM-fixed only has three parameters that can be adjusted to adapt the model to a certain participant’s bioenergetic system, while the 3TM-fixed has eight parameters. The lower number of parameters makes the 2TM-fixed stiffer, which in this case results in larger errors in *E*_*an,acc*_, while the 3TM-fixed is flexible enough to reduce this error. However, this means that the 3TM-fixed model could also be flexible enough to make two or more contradicting sets of estimated parameters give similarly accurate results. In essence, we might be suffering from an indeterminate system, in which case, additional input data is needed for a reliable system determination. This suggests that, for instance, the distribution between phosphagen and lactic energy is established by their interacting effect on the dependent variables, such as *E*_*an,acc*_. Therefore, the increase in one model parameter might be compensated by a corresponding alteration in another parameter. Additional data might be acquired by a testing protocol that makes sure the skier achieves the bioenergetic limits of performance in each STT. For example, a skier has to reach maximal lactic glycolysis rate and the maximal accumulated anaerobic energy expenditure. It would also be beneficial to include periods of more pronounced anaerobic recovery, with zero or very low power output.

### Consistency in Estimated Parameter Values

Ideally, the parameter estimation would have resulted in *M*_*O2*_ of 2TM-free and 3TM-free being close to *MR*_*ae,peak*_ in STT1. Instead *M*_*O2*_ of both the 2TM and 3TM was significantly lower for the “free” compared to the “fixed” model versions. The optimal solution to minimizing the error between modeled and measured *MR*_*ae*_ was shown to be a plateau value of *MR*_*ae*_ equal to the value *M*_*O2*_ (Figure 1B). In order for the error to be minimized, this plateau, and hence the value of *M*_*O2*_, needs to be slightly lower than *MR*_*ae,peak*_.

Morton (1986) argues that *θ* > 0 since light workloads can be maintained without any accumulation of lactate, *θ* < 1−*ϕ* since the onset of lactate production occur at workloads less than $\dot{V}\,O_{2,m\,a\,x}$, and *ϕ* > 0 since a true asymptotic $\dot{V}\,O_{2,m\,a\,x}$ can be observed under severe workloads. Since the O_2_ tank was allowed to reach above the top of the P tank, the equivalent of the first above conditions for the 3TM-free and 3TM-fixed would be *θ* + *ψ* > 0. Though the values of θ and ψ were typically small (∼0.08), all these conditions were fulfilled for all participants and both the 3TM-free and 3TM-fixed models in the present study.

The choice of allowing the O_2_ tank to reach above the A tank (2TM-free and 2TM-fixed) or P tank (3TM-free and 3TM-fixed) counteracts an otherwise certain error at the start of the STT, where the measured aerobic rate naturally is non-zero. However, the parameter estimation adjusted the parameter ψ to a value that minimized the cost function for the whole race, and therefore the estimated aerobic metabolic rate at onset of exercise did not agree with the corresponding measured aerobic metabolic rate (Figures 1B, 2B).

Additionally, Morton (1990) reasoned that *λ* > *ϕ* must be fulfilled based on the “hitting the wall” phenomenon experienced in marathons, where a runner’s glycogen stores are depleted during a prolonged sub-maximal workload. However, since the current study used a short duration exercise, glycogen depletion is very unlikely, and hence the above argument is not applicable. Considering the modeled remaining available capacity of the lactic glycolytic system to represent the accumulation of lactate in the working muscles, it instead seems likely that *λ* < *ϕ* could be possible, since the opposite assumption (*λ* > *ϕ*) suggests that maximal lactate concentration can be attained before reaching $\dot{V}\,O_{2,m\,a\,x}$. In the current study, *λ* < *ϕ* was true for all but one participant in the 3TM-free and another in 3TM-fixed. Also, λ is equal to zero for one participant in the 3TM-fixed, but close to zero in both 3TMs for numerous participants.

Whether the capacities *E*_*P,max*_ and *E*_*L,max*_ reflect available energy have not been clearly defined (Morton, 1986, 1990). However, since all parameters in the current study, including *E*_*P,max*_ and *E*_*L,max*_, were derived from measured data, it is reasonable to assume that the modeled capacities reflect available energy. Consequently, it makes sense to be unable to deplete the lactic glycolytic capacity until the very moment of exhaustion, as is the case if *λ* is zero.

The fact that both *M_L*, *M*_*Lrec*_, *E*_*P,max*_, and *E*_*L,max*_ attained values close to their respective bounds indicate that lower values of RMSE could have been found with parameter values outside the prescribed allowed ranges for the 3TM. In fact, preliminary parameter estimations yielded slightly lower RMSE values for solutions with unreasonable parameter values from a physiological point of view. The generous upper and lower bounds were applied to avoid these physiologically unreasonable parameter values in the final parameter estimations. It was reasoned that as the 3TM was created to mimic the human bioenergetic system, solutions with unreasonable parameter values would be of minor interest, even if they yielded lower RMSE values. The allowed ranges were chosen according to previous physiological findings (see section “Parameter Estimation”) and expanded generously to avoid too limiting restrictions during the parameter estimation. Thus, it is unlikely that any physiologically reasonable parameter values could be found outside of these allowed ranges. However, the fact that more optimal solutions likely could be found with parameter values outside the physiologically reasonable ranges suggests that the fundamental formulation of the 3TM cannot fully capture the dynamics of the human bioenergetic system.

### The Parameter Estimation Method

The relatively low values of RMSE for all models for STT1 imply that the method of parameter estimation of the hydraulic bioenergetic models has been successful in finding optimal parameter values. Thus, the deviations between model and measurement data should rather be considered to originate from the model formulations and/or a non-optimal test protocol, rather than a non-optimal parameter estimation.

## Conclusion

The purpose of this study was (1) to develop a method of individual parameter estimation of the 2TMs and 3TMs and (2) to assess and compare the validity of the models in their continuous prediction of aerobic and anaerobic metabolic energy utilization during sprint roller skiing with arbitrary metabolic power outtake.

Regarding aim (1) of the current study, to develop a method of individual parameter estimation for the 2TMs and 3TMs, the described method of parameter estimation was successfully applied to the models in this study. The limitations discussed can be attributed to the model formulations or experimental data. One major concern is the signs of an underdetermined system when it comes to the 3TM-free and 3TM-fixed. The suggested remedy is to adjust the test protocol to ensure that a single optimal set of parameters can be achieved. Still, among the four bioenergetic models in the current study, the 2TM-free provided the highest validity in *MR*_*ae*_ and *E*_*an,acc*_ for the parameter estimation in STT1.

Regarding aim (2), the assessment of validity and reliability of the models’ prediction of aerobic and anaerobic metabolic energy utilization, results showed that the validity was better for the 2TM-free compared to the three other models. This is demonstrated by lower median RMSE and lower model-to-measurement mean difference in both *MR*_*ae*_ and *E*_*an,acc*_ for STT2. Another main finding was the higher reliability for the 2TM-free compared to the other models investigated in this study, as indicated by the lower typical error in both *MR*_*ae*_ and *E*_*an,acc*_ for STT2. In particular, the RMSE of *E*_*an,acc*_ was much higher in STT2 compared to STT1, but this was probably due to the model assumption of fully recovered anaerobic capacities, which was unlikely to be the case for all participants. Therefore, future investigations are needed to assess the model validity and reliability in further detail.

## Data Availability Statement

The raw data supporting the conclusions of this article will be made available by the authors, without undue reservation.

## Ethics Statement

The studies involving human participants were reviewed and approved by the Regional Ethical Review Board of Umeå University, Umeå, Sweden (#2013-59-31). The patients/participants provided their written informed consent to participate in this study.

## Author Contributions

JL, EA, and DS were responsible for the conception and design of the study, acquisition, analysis, and interpretation of the data, drafting the manuscript, and revising it for intellectual content. EA was responsible for the collection of experimental data whereas JL and DS were responsible for the bioenergetic modeling. All designated authors agreed to be accountable for all aspects of the work and have approved the final version of the manuscript. All authors contributed to the article and approved the submitted version.

## Conflict of Interest

The authors declare that the research was conducted in the absence of any commercial or financial relationships that could be construed as a potential conflict of interest.

## Publisher’s Note

All claims expressed in this article are solely those of the authors and do not necessarily represent those of their affiliated organizations, or those of the publisher, the editors and the reviewers. Any product that may be evaluated in this article, or claim that may be made by its manufacturer, is not guaranteed or endorsed by the publisher.
